# Advances in Biotechnology and the Development of Novel Human Vaccines

**DOI:** 10.3390/vaccines13090989

**Published:** 2025-09-22

**Authors:** Ioanna Papadatou, Athanasios Michos

**Affiliations:** 1 First Department of Paediatrics, National and Kapodistrian University of Athens, “Aghia Sophia” Children’s Hospital, 11527 Athens, Greece; amichos@med.uoa.gr; 2 Immunobiology and Vaccinology Research Lab., First Department of Paediatrics, National and Kapodistrian University of Athens, 11527 Athens, Greece; 3 Infectious Diseases and Chemotherapy Research Laboratory, Medical School, National and Kapodistrian University of Athens, ‘Aghia Sophia’ Children’s Hospital, 11527 Athens, Greece

**Keywords:** biotechnology, novel vaccine platforms, mRNA vaccines, DNA vaccines, VLPs, novel adjuvants, transcutaneous immunization, mucosal vaccines, antigen delivery

## Abstract

Recent advances in biotechnology have fundamentally reshaped the landscape of vaccine development, offering innovative strategies to improve immunogenicity, safety and accessibility. This review explores the cutting-edge platforms—including mRNA, DNA, virus-like particles, viral and bacterial vectors, and bacteriophage-based vaccines—that are redefining how vaccine antigens are delivered to the immune system. We also discuss alternative delivery methods, such as transcutaneous and mucosal immunization, which have the potential to improve vaccine acceptance and distribution, as well as next-generation adjuvants targeting innate immune receptors aiming to further enhance vaccine efficacy, especially in vulnerable populations. By synthesizing these innovations, this review highlights how biotechnology is enabling the design of safer, more efficient, and more adaptable vaccines to address both existing and emerging infectious diseases.

## 1. Introduction

Vaccination remains one of the most impactful public health tools for preventing infectious diseases. The first successful vaccine, developed using a live attenuated smallpox virus, demonstrated the ability to confer durable protective immunity. Subsequently, inactivated, live-attenuated, subunit, polysaccharide, and conjugate vaccines have been used to successfully protect against pathogens that cause significant morbidity and mortality. However, existing vaccines often confer suboptimal protection to high-risk individuals with primary or secondary immune deficiencies and the elderly, while to date, there are no effective vaccines for several pathogens causing significant morbidity and mortality, such as human immunodeficiency virus (HIV), hepatitis C virus (HCV), and tuberculosis [1,2,3,4,5].

During the past decade, significant technological advances in diverse scientific disciplines, including genetic engineering, molecular and cellular immunology, structural biology, bioinformatics, computational biology, nanotechnology, and synthetic biology, have been used for the development of next-generation vaccine platforms [6,7].

These innovative approaches include recombinant viral vectors, virus-like particles (VLPs), messenger RNA (mRNA) vaccines, synthetic DNA-based vaccines, bacterial vector systems, and novel adjuvants, and aim to optimize the targeting of specific antigens and improve immune responses to vaccines. At the same time, alternative routes of immunization that could offer needle-free and/or cold-chain-free vaccine delivery are being investigated in order to replace conventional intramuscular and subcutaneous vaccines.

The wide use of mRNA vaccines during the SARS-CoV-2 pandemic has signaled the beginning of a new era of vaccinology, bridging the empirical vaccine development of the past with the rapidly evolving technology of today.

In this review, our objective is to summarize the recent advances in antigen delivery platforms, novel vaccine adjuvants, and alternative routes of immunization that are set to pave the way to the next generation of vaccines.

A literature search was performed in Medline (PubMed) for English-language articles. No additional inclusion or exclusion criteria were applied. Additional references were obtained by searching citation lists of retrieved articles, and the authors identified further appropriate references for inclusion based on expert knowledge. A systematic search methodology was not used.

## 2. Biotechnological Advances for Novel Vaccine Platforms

### 2.1. mRNA Vaccines

During the COVID-19 pandemic, vaccination with SARS-CoV-2 vaccines was estimated to prevent over ten million deaths globally, largely due to the unprecedented speed at which mRNA vaccines were developed [8]. The straightforward design of mRNA—encoding the antigen and delivered in lipid nanoparticles—enabled a rapid transition from sequence identification to clinical evaluation within just a few months.

Despite this success, the widespread use of mRNA vaccines also revealed certain limitations related to immunogenicity and reactogenicity. With respect to immunogenicity, protection was found to wane relatively quickly across all age groups, leading to a reduced duration of immunity. Furthermore, immune responses were markedly diminished in high-risk populations [9,10,11,12,13].

The safety and reactogenicity profile of the first licensed SARS-CoV-2 mRNA vaccines was initially established in pre-authorization clinical trials. However, large-scale vaccination programs rapidly generated real-world evidence that surpassed the breadth and depth of trial data. National immunization campaigns and independent studies in specific populations and varied vaccination schedules contributed substantially to post-authorization safety monitoring. Overall, these data confirmed a favorable safety profile for mRNA vaccines, while also identifying rare adverse events, such as Bell’s palsy (rare; ≥1/10,000 to <1/1000) and myocarditis/pericarditis (very rare; <1/10,000), occurring predominantly in adolescent and young adult males after the second dose [14,15,16].

mRNA immune reactivity has been proposed as the most prominent mechanism for systemic adverse reactions and myocarditis post-immunization with SARS-CoV-2 mRNA vaccines. It is known that exogenous mRNA is intrinsically immunostimulatory and it is recognized by a variety of cell surface, endosomal, and cytosolic innate immune receptors [17]. This innate immune response to mRNA enhances vaccine immunogenicity, as it can provide adjuvant activity to drive dendritic cell (DC) maturation, and thus elicit robust T- and B-cell immune responses. In this way, mRNA vaccine antigens can serve as both “the antigen” by encoding the viral protein, and “the adjuvant” due to its immunostimulatory properties [18].

In some cases, however, the innate response to mRNA can cause an exacerbated immune response, known as “mRNA immune reactivity,” where DCs and Toll-like receptor (TLR)-expressing cells express cytokines and activation markers that trigger a cascade of hyper-inflammation, causing unfavorable systemic reactions with detrimental effects in different organs, including the myocardium [19,20].

Importantly, the risk of myocarditis and pericarditis following COVID-19 infection remains higher than that observed after vaccination. Given that vaccination prevents substantial morbidity and mortality, the overall benefit–risk balance of SARS-CoV-2 mRNA vaccines remains strongly positive [18].

Looking forward, a deeper understanding of the immunological mechanisms underlying mRNA vaccines, combined with advances in antigen design and delivery systems, is expected to drive the development of next-generation vaccines with broader efficacy. Indeed, mRNA vaccines are currently being explored against a wide range of pathogens, including CMV, RSV, HIV, and influenza [21,22,23].

To address the limitations of first-generation vaccines, a novel platform based on self-replicating mRNA (srRNA) is under investigation. Unlike conventional mRNA vaccines, which only encode the target antigen (e.g., the SARS-CoV-2 spike protein), srRNA vaccines incorporate additional sequences derived from positive-sense single-stranded RNA viruses such as alphaviruses. These sequences enable intracellular RNA replication and amplify antigen expression [24,25,26,27].

srRNA vaccines offer several potential advantages. Because of their self-amplification, they require substantially lower doses to elicit comparable immune responses, which may reduce RNA-associated reactogenicity. Prolonged antigen expression from smaller initial doses could also enhance the magnitude and durability of protection. Immunostimulatory properties may vary depending on the delivery system, the replication machinery itself, and the encoded antigen. Although the addition of self-replicative elements could raise concerns regarding their safety profile, preclinical safety and biodistribution studies have so far reported no major adverse events, aside from transient fever and localized inflammation. In animal models, the highest srRNA concentrations were found in muscle, lymph nodes, and spleen, and remained detectable for up to 60 days after a single immunization [28,29].

Overall, srRNA vaccines represent a promising evolution of mRNA technology, combining potent and durable immune responses with reduced RNA doses. Although their clinical development is progressing more slowly than that of conventional mRNA vaccines, multiple preclinical and clinical trials are underway. Notably, two srRNA-based COVID-19 vaccines have recently received approval in India and Japan [30,31].

### 2.2. DNA Vaccines

DNA vaccines have emerged as a highly promising approach, with potential advantages in terms of stability and ease of manufacturing [19]. Unlike protein-based antigens of subunit vaccines requiring complex purification, DNA constructs are chemically stable and purified through simple procedures. Their modular design allows the expression of single or multiple antigens, and the co-delivery of immunostimulatory molecules such as cytokines as adjuvants. Although several DNA vaccines are already in use for the prevention of animal disease, their integration into human use has been slower [32,33,34].

In 1990s, a preclinical study determined that intramuscular plasmid DNA injection could drive antigen expression without specialized delivery systems [35]. However, these first ‘naked’ DNA vaccines had low immunogenicity, mostly due to rapid DNA clearance. Utilizing various delivery systems such as viral vectors, virus-like particles, and electroporation, as well as selecting effective adjuvants such as CpG DNA, has been an important strategy in improving the immunogenicity of modern DNA vaccines, and has been the focus of current research in this field in order to develop protective human DNA vaccines [36,37,38,39,40,41,42,43]. These approaches have been summarized in the recent literature [44,45].

Apart from the immunogenicity limitations, concerns exist regarding potential genomic integration. Although plasmid DNA is circular and administered in small doses, and thus has a very low integration risk, continued monitoring is necessary [37,38,39,40,41].

The first DNA vaccine to enter a phase I clinical trial was developed against HIV-1 in the late 1990s. While it did not progress to licensure, the trial established proof of concept and alleviated many early safety concerns [46]. Since then, hundreds of DNA vaccines targeting diverse pathogens, including influenza, Zika, HIV, and Toxoplasma gondii, have entered or completed clinical trials [47,48,49,50]. The first DNA vaccine authorized for human use, the ZyCovD vaccine against SARS-CoV-2, received emergency authorization in India during the COVID-19 pandemic, with a 66.6% efficacy reported in the interim analysis of a randomized phase 3 trial [51].

The broader use of DNA vaccines in the future clinical practice is based on the optimization of plasmid design, delivery methods, and immune adjuvants, to maximize antigen expression and stimulate potent and durable immune responses [52,53].

### 2.3. Virus-like Particle (VLP) Vaccines and Synthetic VLP (sVLP) Vaccines

Virus-like particle (VLP) vaccines, synthetic VLP and nanoparticle (NP) vaccines have been developed to deliver antigens as purified proteins, but with increased immunogenicity and stability compared to traditional subunit vaccines.

Virus-like particles (VLPs) arise from the self-assembly of viral structural proteins, which closely mimic authentic viral particles. VLPs are classified into two main categories: nonenveloped and enveloped. Non-enveloped VLPs do not possess an external lipid membrane, while enveloped VLPs are surrounded by a host-derived lipid bilayer that incorporates viral glycoproteins. Both enveloped and non-enveloped VLPs can be either single or multilayered and may be assembled from a single protein or multiple proteins [54,55,56,57,58].

VLPs are considered highly immunogenic, as they induce robust cellular and humoral immune responses due to their highly repetitive display of antigenic epitopes. Licensed VLP vaccines include the hepatitis B vaccine (HBV) and the human papillomavirus vaccine (HPV), which have been widely used, with proven post-licensure safety and effectiveness.

Novel VLP-based vaccines against viruses SARS-CoV-2, influenza A, EBOV, adenovirus-7, HPV, HIV-1, hepatitis B, SARS-CoV, dengue, rabies, rotavirus, norovirus, and malaria have been recently approved or are currently being tested in clinical trials [59].

### 2.4. Viral Vector Vaccines

Viral vector vaccines (VVVs) are engineered by integrating foreign antigen-encoding sequences, typically immunologically relevant transgenes, into the genome of a virus using recombinant DNA technology. Safety is driven by a variety of attenuation strategies to reduce pathogenicity, including the deletion of virulence genes and the incorporation of conditional replication circuits that only function in specialized cells [60].

To date, viral vector platforms have been broadly categorized into two main types: replication-competent and replication-deficient vectors. Replication-competent vectors retain the ability to replicate within host cells, thereby amplifying the expression of the transgene and generating infectious progeny. This replication mimics natural infection, often resulting in a robust and durable immune response. Replication-competent vectors include platforms based on adenoviruses, retroviruses, rhabdoviruses, and paramyxoviruses, whereas replication-deficient systems commonly utilize adenoviruses, retroviruses, and adeno-associated viruses. Additionally, single-cycle viral vectors have been developed, particularly from the adenovirus and rhabdovirus backbones, which allow only one replication round in order to optimize the safety profile of candidate vaccines. In contrast, replication-deficient vectors are engineered to deliver a single copy of the transgene without undergoing replication, enhancing their safety profile. These vectors typically rely on exogenous promoters to drive immunogenicity, but may elicit comparatively weaker immune responses, often necessitating the inclusion of adjuvants to enhance immunogenicity [61,62,63,64,65,66,67].

The large-scale deployment of adenoviral vector-based vaccines during the SARS-CoV-2 pandemic has catalyzed considerable interest in the platform. However, significant safety signals were raised during post-licensure surveillance because of serious adverse events including thrombosis with thrombocytopenia syndrome (TTS). These real-world data led to risk–benefit reassessment and change in recommendations in several countries [68]. Other concerns regarding viral vector vaccines include pre-existing anti-vector immunity, which could limit the immunogenicity of the vaccine. Investigational approaches to minimize the risks associated with VVV include., single-cell replication designs and robust post-marketing surveillance [69]. For adenovirus backbones, serotype exchange and capsid genetic and chemical engineering have the potential to overcome the main safety challenges highlighted by the experience with the COVID-19 vaccines [69].

Numerous ongoing clinical trials are currently evaluating recombinant viral vector vaccines for a variety of pathogens, such as respiratory syncytial virus (RSV), Chikungunya virus, West Nile virus, Lassa fever virus, Zika virus, and Ebola virus [60].

### 2.5. Bacterial Vector Vaccines

During the last decade, several scientific efforts have focused on the utilization of both attenuated pathogenic or commensal bacteria in the development of therapeutic and preventive vaccines. For vector engineering using pathogenic bacteria, the principal idea is to harvest their high immunogenicity while impairing their pathogenicity through gene modification or attenuation. For vectors that use commensal bacteria, the aim is to take advantage of their immunoregulatory and anti-inflammatory properties [70].

An example is Listeria monocytogenes used in cancer immunotherapy against melanoma, delivering tumor-associated antigens [71]. In the past, bacterial vectors of Lactococcus lactis and Bordetella pertussis have been investigated for the development of an HIV vaccine and have provided significant insights on bacterial vaccine technology [72].

Today, several attenuated bacterial species are being extensively investigated as vectors for DNA vaccine delivery, including Yersinia enterocolitica, Listeria monocytogenes, Salmonella spp., and Shigella spp. In parallel, nonpathogenic lactic acid bacteria—such as the Lactococcus, Streptococcus, and Lactobacillus species—have emerged as attractive alternatives due to their safety profiles and mucosal colonization capabilities. Salmonella has been the most extensively investigated vaccine vector strain due to its immunogenic properties. Attenuated Salmonella vectors stimulate robust immune responses due to pathogen-associated molecular patterns (PAMPs) such as lipopolysaccharides (LPS) and flagellin and induce a primarily Th1 response that promotes the development of antigen-specific adaptive immunity [73,74].

Although bacterial vaccines hold significant promise, there are still major concerns about their safety, particularly about the potential for horizontal gene transfer of virulence factors or antibiotic resistance genes [75].

### 2.6. Bacteriophage-Based Vaccines

Bacteriophage-based vaccines have garnered increasing attention as a versatile platform for antigen delivery and vaccine development, leveraging the inherent properties of bacteriophages, which are viruses that infect bacteria, to elicit robust immune responses [76]. Bacteriophages can be engineered to display foreign antigens on their surface (phase-display vaccines), to carry a DNA plasmid encoding the target antigen (phage–DNA vaccines), or both (hybrid phage vaccine).

Although, veterinary bacteriophage vaccines have gained more relevance in recent years, several investigational phage-based vaccines have been developed for human applications in both preventive vaccines against infectious diseases such as Dengue fever, influenza, and SARS-CoV-2 [77,78,79,80], as well as therapeutic vaccines for cancer therapy [81,82,83,84].

Although bacteriophages present a promising avenue for vaccine development, challenges remain in terms of characterizing the phages and optimizing their delivery and immunogenicity [76].

### 2.7. Multi-Epitope Assembly Polypeptide System (MAPS)

The multiple-antigen presenting system (MAPS) is a novel conjugation platform for polysaccharide antigens that aims to overcome the shortcomings of current protein-conjugated polysaccharide vaccines, such as pneumococcal conjugate vaccines (PCVs). MAPS utilizes the high-affinity biochemical interaction between the biotin and rhizavidin in order to assemble strong multiantigen complexes. This technology offers considerable flexibility, allowing for a precise and interchangeable combination of polysaccharide and protein components. Unlike traditional conjugate vaccines, MAPS constructs incorporate pathogen-specific proteins that could potentially induce broad, multiantigen immune responses. To date, MAPS-based vaccine candidates have undergone extensive preclinical evaluation, and one pneumococcal formulation has advanced to early-phase clinical trials in healthy pediatric and adult populations, including older adults [85,86].

MAPSs are designed to contain multiple copies of B cell and T cell epitopes, enhancing immunogenicity. Furthermore, MAPS vaccines could be rapidly designed and manufactured, which makes them particularly valuable for addressing emerging infectious diseases and pandemic preparedness [86].

**Figure 1:**
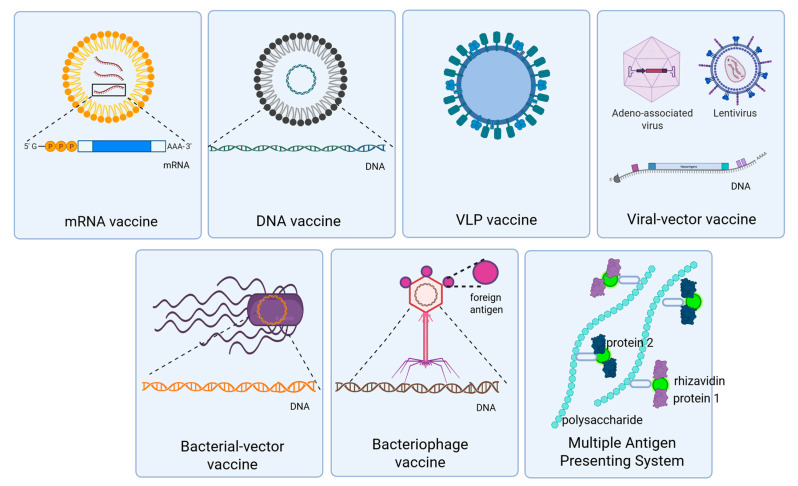
Novel vaccine platforms. Graphical representation of the novel biotechnological platforms utilized in vaccine research pipelines: mRNA vaccines, DNA vaccines, virus-like particle (VLP) vaccines, viral vector vaccines, bacterial vector vaccines, bacteriophage vaccines, multiple-antigen presenting system (MAPS) vaccines. (Created with Biorender).

The constructs of different novel vaccine platforms are summarized in Figure 1. The mechanisms of action, advantages, and disadvantages are summarized in Table 1.

## 3. Biotechnological Advances Towards Needle-Free Vaccine Delivery

In parallel with the significant ongoing work on novel vaccine platforms, another important area of investigational vaccinology is on alternative modes of vaccine administration. Traditional intramascular and subcutaneous injections require time off for medical appointments, administration by a trained healthcare professional, and most importantly, are associated with pain and stress for the vaccinees. Therefore, several approaches for needle-free vaccine delivery are underway, including transcutaneous (TCI) and oral or inhaled mucosal vaccines.

Transcutaneous immunization could offer needle-free vaccine delivery that deposits the vaccine within the epidermal layer of the skin, with the potential to simplify administration and improve vaccine uptake. Different technologies used in TCI include transdermal electroporation, sonophoresis, microneedle patches, skin radiofrequency/thermal and laser ablation, jet or powder injection, and iontophoresis [87,88,89,90,91,92]. Intradermal delivery has been investigated for seasonal influenzas immunization [93] and fractional dose regimens for polio vaccination [94]. The principle of vaccine delivery through skin is based on the activation of tissue-resident immune cells of the innate arm of immunity, such as professional antigen-presenting cell (APC) populations within the skin, various dermal dendritic cells (dDCs), and macrophages that can in turn activate a cascade of robust adaptive T-cell and B-cell immune responses. However, identifying the optimal strategies to penetrate the stratum corneum (SC) of the skin, which serves as the primary barrier to vaccine components, as well as the appropriate antigens and adjuvants for the activation of skin APCs pose significant challenges to engineering safe and highly immunogenic transcutaneous vaccines [93]. In terms of perspective, such vaccination approaches could lead to greater overall compliance to immunizations, overcoming many of the current limitations of traditional vaccines.

Mucosal vaccines, administered via the nasal, oral, or pulmonary routes, for respiratory and gastrointestinal pathogens are another important field of ongoing vaccine research. Similarly to TCI, the induction of an immune response by mucosal vaccines is based on the activation of tissue-resident immune cells that can in turn activate a systemic adaptive immune response to vaccine antigens [95]. Mucosal vaccines, delivered orally, nasally, or through other mucosal routes, encounter specialized antigen-presenting cells such as dendritic cells and macrophages. These APCs reside in organized mucosal-associated lymphoid tissues, such as Peyer’s patches in the gut and nasal-associated lymphoid tissue in the nasal passages and can in turn activate antigen-specific T-cell and B-cell responses. Licensed mucosal vaccines include the oral rotavirus and polio vaccines, as well as the inhaled live attenuated influenza vaccine.

However, development of mucosal vaccines has been significantly slower than that of the traditional injectable vaccines. The major obstacles for the development of effective mucosal vaccines are our gaps of knowledge regarding protective mucosal immunity and the lack of set correlates of protection for mucosal immunity; the susceptibility of unprotected subunit antigens to degradation and clearance, and crucially, a lack of effective mucosal adjuvants [95].

New mucosal pneumococcal [96,97] and SARS-CoV-2 vaccines [98] are currently being investigated, in order to develop immunity against pathogen acquisition and carriage of pathogens, which could significantly increase herd immunity and minimize transmission of these pathogens compared to current licensed vaccines.

## 4. Biotechnological Advances for Slow Antigen Delivery

Effective vaccine-induced immunity has proved difficult for several rapidly mutating pathogens, including HIV-1. The amount of somatic hypermutation associated with the development of broadly neutralizing antibodies against HIV has not been achieved using conventional immunization strategies. An underexplored aspect of vaccine design is modulation of antigen kinetics.

Extended antigen delivery through continuous immunization over several days has recently been shown to significantly enhance the immune response to HIV antigens. A slow release immunization over two weeks using nonmechanical osmotic pumps (OPs) and a soluble adjuvant resulted in enhanced BGC and TFH cell responses in mouse models [99,100]. Similarly, slow delivery immunization of rhesus monkeys (RMs) resulted in more robust T follicular helper (TFH) cell responses and GC B cells with improved Env-binding.

The potential mechanisms include shifting B cell recognition away from non-neutralizing immunodominant epitopes, altered kinetics of immune complex deposition, improved T follicular helper (Tfh) cell responses, enhanced affinity maturation, and enhanced development of B cell memory. Long-term antigen depots may have broad utility to improve antibody responses to vaccines, offering several advantages over conventional (bolus) immunization strategies [100].

The development of clinically applicable slow delivery technologies is an active area of research and holds promise for improving vaccine efficacy, especially for challenging targets such as HIV. These technologies could include degradable encapsulating biomaterials and depot-forming adjuvants that slowly release antigens over time, maintaining germinal centers and enhancing B cell maturation [101].

## 5. Biotechnological Advances for Novel Vaccine Adjuvants

Another key aspect of the engineering of more efficacious vaccines is the design of novel vaccine adjuvants that could multiply the immunogenicity of non-live vaccines [102,103].

Vaccine adjuvants are important components of a number of ‘traditional’ subunit vaccines in order to achieve optimal immune responses. Although certain novel vaccine platforms are intrinsically immunostimulatory (e.g., mRNA-LNPs and viral vectors), others like DNA vaccines and VLPs require the use of additional adjuvants in order to increase immunogenicity, lower antigen dose, and/or tailor responses for specific high-risk populations [103].

The adjuvants currently licensed were developed mostly empirically and the design of novel more effective adjuvants is based on increased understanding of innate immunity and antigen-presenting cells and the utilization of biotechnological tools. For several decades, beginning in the 1920s, insoluble aluminum salts were the first and only adjuvants used to improve vaccine efficacy against infectious diseases. Since then, several additional adjuvants have been included in approved vaccines [104]. The most widely used adjuvant, alum enhances antibody responses, but is less effective at inducing cellular immunity. Its mechanism of action is still being investigated but likely involves creating a depot at the injection site, enhancing antigen uptake, and activating the NLRP3 inflammasome [105].

Research for novel vaccine adjuvants has increasingly been focused on targeting innate immune sensors—particularly Toll-like receptors (TLRs)—to amplify both cellular and humoral responses. Identification and functional characterization of pattern recognition receptors (PRRs) have significantly advanced the development of vaccine adjuvants targeting the innate immune system. Several PRR agonists are now licensed for clinical use or in late-stage preclinical development, offering novel tools to improve vaccine immunogenicity, particularly for populations with suboptimal immune responses [105].

One of the most promising such adjuvants is monophosphoryl lipid A (MPLA), a detoxified derivative of Salmonella minnesota lipopolysaccharide, and a TLR4 agonist. Unlike native LPS, MPLA retains robust immunostimulatory properties while exhibiting a ~1000-fold reduction in reactogenicity [106]. TLR9 agonists, particularly CpG-containing oligodeoxynucleotides (CpG ODNs), mimic bacterial DNA motifs. These agonists activate TLR9-expressing plasmacytoid dendritic cells, B cells, and NK cells, enhancing humoral and Th1-cellular immune responses [107]. Heplisav-B, a licensed hepatitis B vaccine containing a CpG adjuvant, has shown superior immunogenicity in adults aged 40 to 70 years compared to conventional alum adjuvant vaccines [108]. TLR7/8 agonists, such as the imidazoquinoline compound resiquimod (R848), activate plasmacytoid and myeloid dendritic cells, up-regulating costimulatory molecules and inducing type I interferons and IL-12. Although their clinical application has been limited by rapid systemic clearance, advances in formulation strategies, including lipid conjugation and nanoparticle encapsulation, have improved their retention and efficacy [109]. Ongoing research is exploring adjuvants that target additional PRRs, including TLR3, TLR5, C-type lectin receptors and RIG-I-like receptors. Table 2 summarizes selected prominent vaccine adjuvants discussed.

The main concern regarding novel adjuvant development is the potential associated increased reactogenicity. Current research focusses on immunoengineering, novel delivery system, and systems biology to identify biomarkers of safety and adjuvanticity [105].

## 6. Discussion

Advances in biotechnology have enabled the development of novel vaccine approaches that leverage a deeper understanding of the pathways and mechanisms of the immune system [60,62].

The COVID-19 pandemic accelerated the clinical deployment of mRNA platforms and catalyzed global investment in adaptable and scalable vaccine technologies. Beyond mRNA, diverse antigen delivery systems are advancing into clinical pipelines, offering novel routes to improve the immune response and induction of immunological memory, promote compliance, and enable personalized vaccination strategies for diverse populations.

However, significant challenges remain regarding safety, reactogenicity, and real-world efficacy. For mRNA vaccines, high effectiveness and agility are tempered by waning immunity and reactogenicity; srRNA may address dose and durability of immunity concerns. DNA vaccines offer stability and simple production but require specialized delivery and the addition of adjuvants in order to increase immunogenicity in humans. Viral vectors provide strong immunity but necessitate vigilant safety monitoring and design refinements, as highlighted by rare TTS following adenoviral COVID-19 vaccines. Other novel platforms, such as bacteriophage vaccines, are still in the early stages of clinical implementation into human use.

Needle-free routes of immunization promise improved acceptance and herd effects but must overcome significant limitations regarding skin barrier penetration, understanding of the tissue-specific immunity, and appropriate antigen and adjuvant selection for each route of immunization. The development of novel adjuvants guided by systems vaccinology could be key to tailoring responses across the less immunogenic vaccine platforms as well as mucosal and transdermal vaccines.

Continued interdisciplinary collaboration, supported by mechanistic insights into host–pathogen interactions and immune modulation, will be essential to realize the full potential of these technologies in difficult-to-target pathogens and global pandemic preparedness. Future vaccines will likely combine multiple innovations in terms of vaccine platform, antigens and adjuvants included, as well as delivery strategies in order to provide robust and long-lived protection with acceptable reactogenicity profiles. Equitable access, scale-up, and global pharmacovigilance will determine the real-world impact of these innovations.

## 7. Conclusions

Biotechnological innovations in vaccine platforms, adjuvants, and delivery routes are transforming the landscape of immunization, offering opportunities for more durable, broad, and accessible protection. Future vaccines could utilize several of these advances to achieve safe, long-lasting immunity and improved preparedness against future epidemics.

## Figures and Tables

**Table 1:** Novel vaccine platforms, mechanisms of action, advantages, and disadvantages.

Platform	Mechanism	Advantages	Disadvantages
mRNA vaccines [8,9,10,11,12,13,14,15,16,17,18,19,20,21,22,23]	LNP-encapsulated mRNA-encoding antigen	Rapid design and scale-up; adaptable; elicits humoral and cellular responses	Waning immunity; impaired responses in high-risk groups; reactogenicity; rare serious adeverse events; need for cold chain
Self-replicating mRNA vaccines (srRNA) [24,25,26,27,28,29,30,31]	Encodes replication sequances for intracellular RNA amplification	mRNA dose-sparing; prolonged antigen expression; potentially enhanced durability of protection	Theoretical risk of recombination; heightened innate activation
DNA vaccines [32,33,34,35,36,37,38,39,40,41,42,43,44,45,46,47,48,49,50,51,52,53]	Plasmid DNA-encoding antigen	Simple manufacturing; favorable safety profile	Lower immunogenicity in humans; delivery/electroporation logistics; theoretical risk of integration
VLP vaccines [54,55,56,57,58,59]	Self-assembly of viral structural proteins, closely mimicking viral particles	Highly immunogenic; repetitive epitope display; proven safety and immunogenicity	Difficult assembly for complex antigens; challenging stability and scale
Viral vectors (replication- competent/ single-cycle) [60,61,62,63,64,65]	Vectors replicate or undergo one cycle to express antigen	Robust, durable responses mimicking infection	Increased safety monitoring; rare serious AEs (e.g., TTS for some adenovectors)
Viral vectors (replication- deficient) [66,67,68,69]	Recombinant non-replicating vectors	Improved safety profile	Pre-existing anti-vector immunity could impair immunogenicity; may need higher dose/adjuvant
Bacterial vectors [70,71,72,73,74,75]	Attenuated pathogens or commensals	Induction of mucosal/systemic immunity; immunoregulatory properties	Risk of gene transfer, reactogenicity
Bacteriophage-based vaccines [76,77,78,79,80]	Phage display or phage–DNA hybrid vaccines	Favorable safety profile, potential low manufacturing cost; potential for multivalent display	Limited clinical data in humans; need for optimization of display/folding
MAPS [85,86]	Biotin–rhizavidin assembly of polysaccharides/ proteins	Modular, multi-epitope; broad responses	Manufacturing optimization; immunogenicity data based on comparison to existing conjugate vaccines

**Table 2:** Selected adjuvants, mechanisms of action, advantages and disadvantages.

Adjuvant	Mechanism	Advantages	Disadvantages
Aluminum	Depot effect; NLRP3 inflammasome engagement	Extensive safety record; strong humoral responses	Weak cellular immunity; limited Th1immune response
MPLA (TLR4)	TLR4 activation with reduced toxicity	Balanced Th1/Th2 immune response	Reactogenicity risk; limited licensed products
CpG ODN (TLR9 agonist)	Activates dendritic cells and B cells	Th1 bias; high immunogenicity	Reactogenicity risk; autoimmunity concerns monitored
TLR7/8 agonists	Dendritic cell activation; IFN/IL-12 induction	Potent antiviral responses	Rapid clearance; toxicity at high doses

## Data Availability

Not applicable.
